# A window to the past and future aquaculture in the Gulf of California: the abundant times of ‘Meyibó’

**DOI:** 10.1098/rstb.2024.0041

**Published:** 2025-07-10

**Authors:** Mariana Walther Mendoza, Heidi K Alleway, Sebastián Quiñones, Jonathan Mackay, Giovanni Fiore Amaral

**Affiliations:** ^1^ The Nature Conservancy, Mexico; ^2^ Provide Food and Water, The Nature Conservancy, USA; ^3^ Independent Scholar, Chile; ^4^ Independent Scholar, Mexico

**Keywords:** functionally extinct, pearl oyster beds, shellfish reefs, restorative aquaculture, historical, aquaculture

## Abstract

The Gulf of California has been widely recognized as a global marine biodiversity hotspot. For centuries, the use of aquatic resources in the region has provided food and employment to different groups of people and communities. With demand for seafood continuing to grow, domestically and globally, the pressure on these resources will increase. This raises questions about how community, government and industry can balance socioeconomic and ecological demands, including the need to meet multiple development goals, such as conservation and biodiversity targets, mitigation of greenhouse gas emissions and greater equality. This article describes the long-term impact that different human cultures have had along the coast of the Baja peninsula, with a focus on bivalve species that once created productive and critical habitats such as pearl oyster beds and reefs. Like many places worldwide these habitats are now considered to be functionally extinct and greater intervention through aquaculture and restoration is needed to enable their recovery. Using geospatial analysis that includes historical data from written and verbal sources, we developed and applied a spatial dataset and prioritization process to inform restoration and ecologically sustainable aquaculture development for bivalve species going forward.

This article is part of the theme issue ‘Shifting seas: understanding deep-time human impacts on marine ecosystems’.

## 1. Introduction

The extensive evidence of utilization and exploitation of shellfish reefs shows that these ecosystems and species used to be far more widely spread than they are today [1,2]. In the past, shellfish reefs were likely one of the dominant structural and ecological components of coastal ecosystems in the Baja peninsula, Mexico, with native bivalve species forming extensive communities and associations, consolidated oyster beds and fringe reefs miles in length [1,3]. These ecosystems were comparable to those formed by the extant species *Crassostrea virginica* and *Crassostrea gigas* along the West Atlantic and Pacific Oceans [4]. Biogenic bivalve reefs, which can have increased species richness and abundance in comparison to other nearby habitats [5], are known to play a vital role in supporting, regulating and providing a wide range of ecosystem services such as food and habitat for other species, fish production, water filtration, nutrient cycling, shoreline protection and stabilization, as well as food for human consumption and opportunities for recreation and tourism [6,7]. Unfortunately, these systems are especially sensitive to anthropogenic impacts, such as fishing, eutrophication and the effects of climate change [8,9]. This means current conservation approaches, continuing industry activities and ambitions for further development could be exacerbating past impacts, or overlooking critical needs for the protection of native species and reef ecosystems. Historical and traditional ecological information has enormous potential to assist better decision-making in social-ecological systems, including choices around the types of natural resource management that balance the needs of nature and people in the current environment [10]. Including historical information and appreciation of past peoples’ connections with the sea, can help to identify baselines that are more appropriate to a local context, and more equitable and reflexive under a future of ongoing environmental change [11]. This information can be used to motivate and guide effective restoration work and ecologically sustainable development of aquaculture [12].

Located in northwest Mexico, the Baja peninsula measures around 1200 km in length and extends through two states, Baja California and Baja California Sur. It is surrounded to the east by the Gulf of California, one of the most biodiverse marine systems in the world [13,14], and to the west by the nutrient-rich Pacific Ocean. The region hosts unique and diverse habitats, and dynamic oceanographic processes, such as gyres, strong tidal mixing and wind-driven upwellings, making it one of the most important fisheries and aquaculture regions, contributing over 70% of national landings [14]. Yet, despite its social, economic and environmental importance, the Gulf of California has long been subject to overfishing and coastal development, resulting in habitat loss and significant declines in marine resources, and increased risk to the threats of climate change [15].

The Baja peninsula has also long been a source of curiosity and inspiration for explorers and missionaries, pirates, scientists and travellers; evidence from these early inhabitants and visitors reveals a completely different seascape from what we see today [3]. Historically, bivalves played an important ecological role, but also a social and economic one. Shellfish reefs sustained and fuelled coastal economies for centuries, since the Californio Indigenous groups, descendants from the first settlers who are thought to have migrated to the Baja peninsula tens of thousands of years ago [16–18], such as Pericúes, Guaycuras and Cochimíes. The latter termed ‘Meyibó’ to be the pitahaya season, from June to August, due to the abundance of this valuable fruit, but also other fruits, seeds and mollusc harvest, making it even more appreciable since it was preceded by a season of scarcity and hunger [17,19]. Despite the arid and isolated environment, these groups were able to thrive due to the profound ecological knowledge that allowed them to understand and use their limited resources and the seasons sustainably, including shellfish as an important source of protein, but also as ornaments or artefacts and in burials [20–23].

Pearl oysters are cited as a primary reason why Indigenous and European cultures established contact in the first place [24]. An expedition sent by Hernán Cortés which arrived on the peninsula in 1533 is cited as the first contact with native groups [22]. Since then, stories about a paradisiac island of pearls, abundance and wealth sparked interest in European travellers and the Spanish crown, which coordinated multiple attempts to conquer and colonize this thought-to-be pearl island. The closest colonization effort was through Jesuit missionaries that occupied the Baja peninsula from 1697 to 1767 and managed to establish a chain of 17 missions along the arid and rugged land [25].

As the pearl fishery in the Baja peninsula developed, fishing effort increased; first through ‘armadores’ or shipowners that hired groups of divers, mostly natives who would free dive for these resources [26]. They would be established in the nearest freshwater source to the pearl oyster beds, referred to as ‘placeres’, for the months of harvest [19]. Overexploitation concerns had been documented since 1769, and despite several regulations, including a control system based on pearl fishing licenses imposed by the Spanish crown as early as 1586 [22,27], a prohibition of the pearl fishery decreed by Jesuit missionaries and a reduction in the fishery quota or number of oysters collected in 1857, such regulations required surveillance that was lacking in the region [28]. The fishery intensified with the establishment of several enterprises and exclusive rights or concessions for harvest, in addition to the introduction of the diving helmet, which would allow divers to extend their immersion time and depth [29].

As a result of these declines, the culture of pearl oysters became a focus in the region, with the Baja California Shell and Pearl Breeding Company, S.A., as the first aquaculture endeavour on Espiritu Santo Island from 1904 to 1914. This company successfully developed the cultivation and repopulation of pearl oyster banks (*Pinctada mazatlanica*) as well as the commercialization of pearls and shells; however, it was abandoned due to socio-political conflicts [30]. Pearl oyster beds were decimated, and a closure of the fishery was decreed in 1939 [29]. Although aquaculture is not new in Mexico, initially it was not considered a commercial activity but rather an effort to increase animal protein in rural zones and create job opportunities [31]. It was not until a few decades ago that aquaculture was recognized in the national legal framework and began to be developed as an alternative agricultural activity that has evolved on a commercial scale for a few species [32]. In general, shellfish aquaculture in the Baja peninsula has shown limited development; besides pearl oyster, focus has been mainly on Pacific oyster, also often referred to as the Japanese oyster (*Crassostrea gigas*) and shrimp, introduced during the 1970s; the development of abalone, penshell, mussels and clams during the 1980s; and marine finfish during the 1990s [33].

From 2010 to 2020, the national production of aquatic products in Mexico has had an average increase of 2% in volume (2% for wild-caught fisheries and 4% for aquaculture) and 11% in value (9% for wild-caught fisheries and 13% for aquaculture) [34]. Mexico is now the fourth largest producer of bivalves in Latin America focusing on the production of the Pacific oyster. Although this species has shown stability and economic feasibility, there is an enormous opportunity and potential to diversify and culture native species [35–37]. Additionally, only Baja California and Baja California Sur hold over 55 and 90% of oyster and clam leases at a national level, respectively [38], highlighting that there is installed capacity, technical expertise and infrastructure to stimulate this industry sustainably. Most aquaculture leases in the region currently permit farming of Pacific oysters and other bivalve species, with a few exceptions for fish and seaweed. However, the bulk of these leases is concentrated within the Magdalena Bay lagoon complex on the western side of the peninsula, mainly due to past government support programmes that had a strong focus on rural areas and mariculture of bivalve molluscs, recognizing the high potential for this activity [32]. Also, the majority of current production is contributed by only a few farms due to reported challenges in seed supply, technical capacity, financing and markets, among others [32,39].

Wild pearl oyster and other shellfish reefs and beds in the coastal Baja peninsula are now considered functionally extinct (with more than 99% loss of the original area), found only in dispersed patches and lacking any significant ecological role [1]. They have been subject to overharvest and exploitation for centuries [3]. Going forward, anthropogenic impacts and the effects of climate change, such as changes in sea surface temperature (SST), ocean acidification and ocean deoxygenation which pose serious risks to bivalves, could result in increased propensity to disease and harmful algae blooms, a higher mortality rate in larvae and juveniles and risk of mass death, continuing to challenge their natural recovery [40]. In this work, we analyse historical information on bivalve distribution from a range of published and verbal sources, including scientific, grey literature and explorer journals, and living knowledge through participatory processes, to better understand the importance and predominance of shellfish reefs in the past and present-day around the Baja peninsula. This information is intended to be used within the context of restoration and aquaculture development, specifically approaches to restorative aquaculture, for native species of bivalves. Understanding the distribution, abundance and utilization of these species in the past provides a glimpse of what the future could look like if we invested capacity and resources into appropriate efforts for their recovery, alongside sustainable opportunities for food and livelihoods. We developed this information into a synthesized geospatial dataset. This data can be used to support decision-making and management strategies to maximize ecological benefits and minimize costs.

## 2. Methods

We identified and then reviewed sources of data using a scoping review process, to identify likely accessible sources (e.g. reports, letters, databases or datasets) of historical information, followed by a systematized review of these sources to extract qualitative and spatial data on the location, time period and abundance of native shellfish species. This mixed methods process was used to ensure as much information as possible could be included within our analysis, in particular, the use of written descriptions alongside historical spatial information and present spatial records through participatory processes.

Sources identified in our scoping review included travellers' diaries, journals, government reports, books and historical records, and global and local databases of past and present bivalve distribution. The historical data acquisition of bivalve distribution and abundance in the Baja peninsula had two main components: data that were downloaded and acquired through different stakeholders, and data that were digitized from online resources. We categorized the information collected into three main time periods: historical information (prehistoric to industrial revolution—1760), a late modern period (1760−1950) and contemporary history (1950 to present). Given the types of data available within each period were considerably dissimilar, we used different approaches for collating and analysing information that were most likely to (i) enable the spatial extent of bivalves to be mapped; and (ii) provide contextual information that could inform the abundance of native species across this range and the type of habitat they provided.

### (a) Historical information (prehistoric to 1760)

We reviewed information and databases, targeting areas with prehistoric periods and collating fossil material from stratigraphic sections in the Baja peninsula. The prehistoric data were obtained from the Paleobiology Database (PBDB) [41] navigator app. Using the filters, we selected the class of ‘Bivalvia’, and visualized data for the Mesozoic and Cenozoic eras. This mapped data contained latitude and longitude for each finding and was saved and downloaded as comma-separated values (CSV) for each era and extracted as individual datasets. We applied filters by state, and data were clipped to the Baja peninsula to also include areas that did not specify a state but were spatially within the study area. We linked the CSV files for the two eras selected to add the total species and genus. Using ArcGIS Pro version 3.2.0, the spreadsheet was spatialized by using the latitude and longitude coordinates that could be incorporated with or displayed alongside other geospatial information. The accuracy of this spatial information did not enable precise analysis of distribution because it is associated with samples collected through archaeological excavations, although they provide the location where these species were reported to be found.

### (b) Late modern period (1760–1950)

Information was obtained from books, reports, historic documents and online resources that provided two main types of data: georeferenced maps and descriptions of locations related to pearl-oyster reefs. Maps were found to detail the location of pearl oyster beds mostly with good accuracy. When maps were not available, but descriptions were, we digitized areas or polygons to whichever resolution possible using the qualitative descriptions provided. It was not easy to find earlier resources online as most of these types of books are in physical form and in specific libraries, universities or national archives around the world, and have not been digitized. Sources also required rigorous reading to find the information that was being searched. Thirdly, we identified challenges with the accuracy of these findings, related to the descriptions of locations of bivalve banks. For this reason, the descriptions were classified into three categories: Vague (narrative that mentions the location of the pearl oyster beds in a general area, i.e. ports, bay and coastline), Somewhat Vague (descriptions of places and locations along with better accuracy giving details like ‘located 1 to 2 miles next to the coast of’ or ‘located south of San Lorenzo canal’) and Specific (slightly more precise as they mention that pearl oyster beds could be found from ‘X’ latitude to ‘Y’ latitude or from port ‘A’ to port ‘B’). The ‘Specific’ category was the only one that was digitized. This process was made through ArcGIS Pro by creating a new shapefile as a polyline feature, adding a base map (i.e. Imagery WGS84) and drawing the locations based on the descriptions.

### (c) Contemporary history (1950 to present)

To collect information for present distributions we used information elicited from participatory meetings and official information from fisheries and aquaculture authorities.

#### (i) Participatory meetings

We conducted two participatory mapping exercises, as part of broader workshops associated with building understanding and capacity for aquaculture spatial planning, with representation from the Baja peninsula aquaculture industry, state government, non-profit organizations, community leaders and academia. Our aims in using this participatory approach were to gather insights from industry and community about where they understood shellfish beds to currently exist and to have existed in the past. Participants were asked to record this information as either a point on a map or as an area, and to provide explanatory details including the species (if known). Associating descriptive information with a specific point, polyline or polygon enabled us to gather both spatial data and information regarding the characteristics of shellfish species distributions and habitat through time, including their potential cultivation in the current environment. Like local ecological knowledge, local spatial knowledge emphasizes place-based understanding, and has the benefit of engaging experts as active participants [42].

The first meeting was in La Paz in February 2023 with a hybrid mode, including 29 participants in-person and 14 participants online via Zoom. In-person participants were divided into four groups and were provided two paper maps downloaded from QGIS ESRI National Geographic database using a 1:38 00 000 scale, one for each state (Baja California and Baja California Sur). Participants were asked to draw the areas that they considered appropriate/inappropriate for culturing bivalves. When adding areas to these maps participants were asked to justify or explain the reasons behind their site selection. Each group had a facilitator documenting this information. For the virtual participants, a comparable approach using three break-out groups was used with online collaborative and real-time mapping using the app ‘Felt’, a Google mapping tool that enables multiple participants to draw online. The second mapping exercise was conducted in Ensenada in April 2023, as part of an aquaculture forum organized by the Instituto Mexicano de Investigación en Pesca y Acuacultura Sustentables (IMIPAS, Mexican Institute of Research for Sustainable Fisheries and Aquaculture), with over 220 registered participants. In this workshop, only the in-person mapping was run, with approximately 60 participants, divided into four groups according to field: government, academia, producers and non-profit organizations. We followed the same process used in the first workshop, requesting the same information from participations, but with an additional category to enrich the historical information, this being areas that participants specifically identified as historic sites for bivalve species; whether currently present or locally extinct. Where possible, participants were asked to document the species groups; however, no specific historical dates were provided.

Results from both mapping exercises were compiled, integrated and digitized, including the paper maps and those produced via Felt. Data hosted in Felt was downloaded in a GeoJSON format and opened in ArcGIS Pro, where it was transformed into shapefiles in the forms of points, polylines and polygons depending on the nature of the data. Spatial data were filtered (i.e. group similar data, delete unnecessary information and merge where needed, and it was portrayed asa maps), translated to English, and filtered, considering only those attributes that might give a description of whether there is existing aquaculture, type of species and/or if they were suitable zones. The last step was to merge any layers that had similar topologies. For instance, when exporting features from Felt maps, there were a few types of lines but for the final shapefile, those lines needed to be combined into a single-line shapefile.

#### (ii) Official information from fisheries and aquaculture authorities

We included information on bivalve fishery and aquaculture leases (permits and concessions), considering that these are areas where it is possible to culture groups of species based on their natural occurrence in the wild or their tolerance ranges. Aquaculture data were downloaded from Transparencia Acuícola platform [43], a national database from the Comisión Nacional de Pesca y Acuacultura (CONAPESCA, National Fisheries and Aquaculture Commission) in kmz formats of Google Earth and were transformed into shapefiles in ArcGIS Pro.

Fishery permits were formally solicited from CONAPESCA [44]. For permits that included geographic coordinates, these were used to create polygons that represent the fishing areas, and for permits without coordinates, we used the landing site as the geographic reference. Data were transformed into shapefiles in the forms of polygons and points, in the case of coordinates or landing site, respectively, depending on its nature. The spatial data were filtered based on the species or group of species included in each permit. Furthermore, in the case of polygon shapefiles, these were converted into points by calculating the centroid of each polygon. Finally, these types of permits were merged into a single shapefile so it could be visualized as clusters of points.

## 3. Results

Identifying past and present baselines enabled us to consider how the spatial extent of bivalve ecosystems has changed through time to inform contemporary and future decision-making for natural resource management and conservation efforts. By bringing together qualitative and spatial information across three distinct time periods we were able to compare how bivalve abundance and distribution of oyster reefs and pearl beds has changed along the Baja peninsula.

### (a) Historical information (prehistoric to 1760)

**Figure 1.**
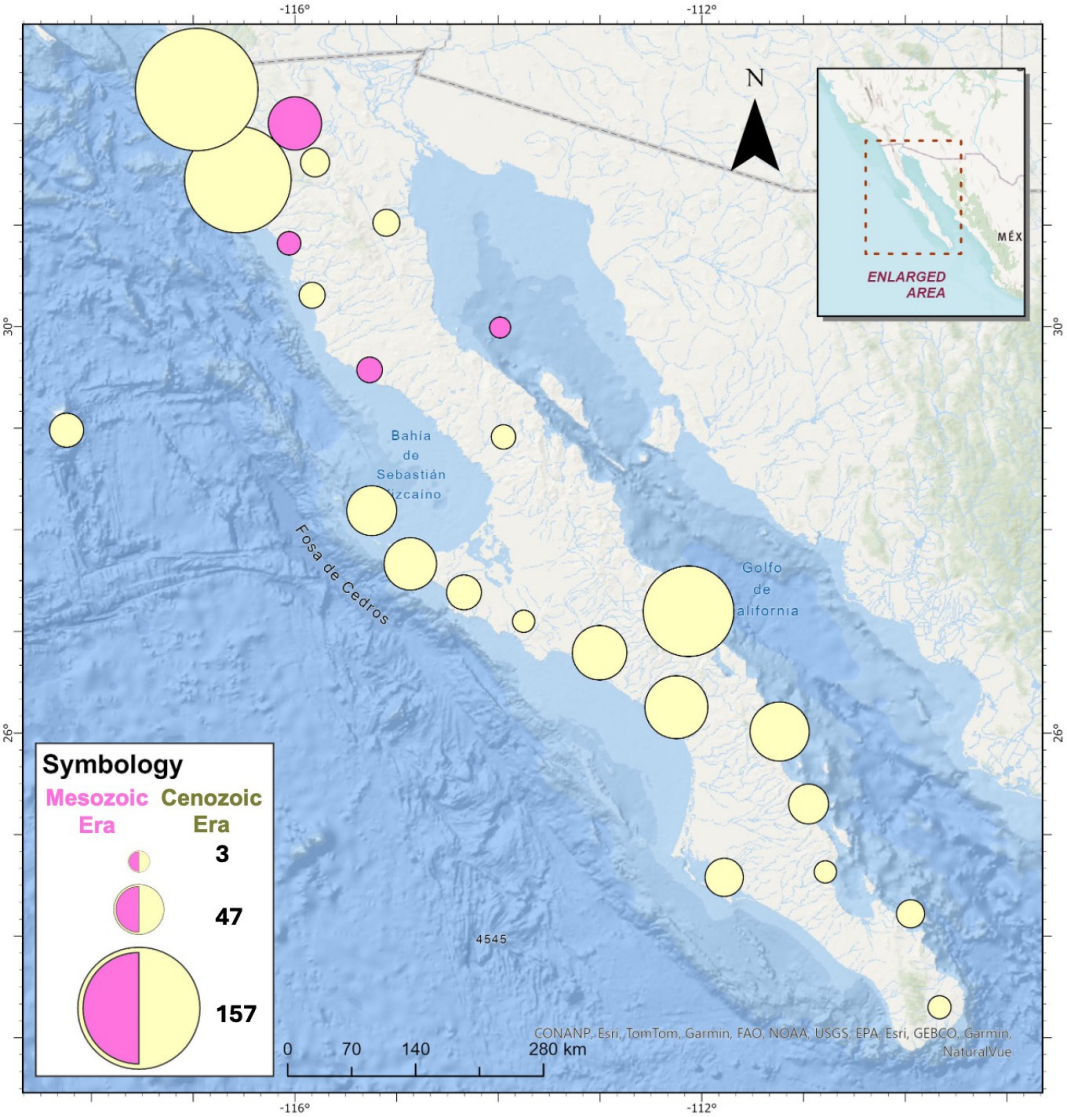
Presence of bivalve fossil records. Records of species and genus of bivalves from the Mesozoic and Cenozoic eras, registered in the Paleobiology Database.

On the Baja peninsula, bivalve fossil records indicate their presence since the Mesozoic Era, during the Late Triassic period, more than 66 Ma [45], and more prominently during the Cenozoic Era, over 23 Ma [46–48]. Since the Late Triassic period and through the Middle Pleistocene, over 265 species (53 for the Mesozoic Era and 212 for the Cenozoic Era) and 225 genera (71 for Mesozoic Era and 154 for the Cenozoic Era) of one class, Bivalvia, have been recorded in the Paleobiology Database for the Baja region (figure 1).

Multiple records and documentation that describe the seascape in the Gulf of California focus on pearl oyster beds due to the historic importance of this fishery, but they also describe the abundance and distribution of other shells and bivalve species. Shell fishhooks manufactured from mussels and abalone found in Isla Cedros, off Baja California, date from the Late Pleistocene [49]. Clavijero [50], Jesuit priest and historian, wrote about the numerous abalone shells in the Pacific, Pacific thorny oyster and pen shell abundance in the Gulf of California. Archaeological studies mention numerous and abundant shell middens with a variety of shell types and of great extent, noting that several attained a mile in length in some sites [20]. Some of the shell mounts date up to 6100 and 7200 years ago, such as clam and mussels, respectively, and some studies suggest that the main species used by the Californio Indigenous groups were chione clam (*Chione sp.*), catarina clam (*Agropecten ventricosus*), chocolata clam (*Megapitaria sp.*) and mule’s paw (*Anadara sp.*), due to their abundance in shell excavations and their ease of harvest [17].

Table 1 compiles a list of citations and quotes from travellers and explorers, soldiers, researchers, buccaneers and missionaries covering a period from 1596 to 1911. Some of these quotes include references to abundance and how the seascape looked in the past, while others mention the weight of the pearls, their size or price. For example, Alexander Taylor stated that a single pearl that was number one in size and lustre was worth $5000−6000 and mentioned that an American minister in 1863 described the pearls as large as a pigeon’s egg and recognized that pearls from the Gulf of California were among the most valuable jewels in the Spanish regalia [51]. Lieutenant R. W. H. Hardy was commissioned by the General Pearl and Coral Fishery Association of London to find beds of pearl oysters from 1825 to 1828, noting in his report that ‘The fishery of Loreto appears to be more productive than that of Panama…’ . Anecdotal information also highlights a rapid decimation of pearl oyster beds through this time period, raising from some of the authors a greater abundance and richness in the past and raising concern about the future of this resource.

**Table 1.** Quotations related to the use and character of pearl oyster beds in the Gulf of California.

anecdote/quote	date	source	written by
‘At this place fish of all kinds were found in such abundance that boats could be loaded with very little labor, and pearl oysters strewed the shores in such unaccountable quantities as to make the beach appear like an immense pavement of brilliant mosaics …’	1596	[51]	Alexander S. Taylor
‘this bay is about four leagues long and the Indians took us to a pearl-bed inside the bay; the pearl-bed is about two leagues in length and four to eight fathoms in depth …native divers got oysters and pearls of different types …’	1633	de-Ortega 1636 (1970) from [3].	Spanish captain and explorer
‘there are quantities of pearl oysters and gold and silver mines which are not exploited.’	1688	[52]	unidentified buccaneer
‘the situations of the beds of oysters I have marked upon the charts (which were likewise sent to me) with a slight shade of carmine. That off the island of Tiburon is considered an excellent lay; but has seldom or never been wrought, on account of the fierceness of the Indians who inhabit the island. But I, who have been accustomed, in the service of the house, to the Malays, would have no objection to try my luck amongst them, for they cannot possibly be worse. Those off Point Lorenzo and the island of Cerrebro, are considered the most productive that are frequented at present, and where they have all been fishing this year. The harbours of Pichiluigo and La Paz are very productive also. In fact, the whole bay of La Paz, and all along the coast as far as Loreto, is the general fishing-ground.’	1829	[53]	Lieut. Robert William Hale Hardy, British navy officer
‘at the end of the XVI century in which "marine mines" were discovered, inhabitants of New Galicia, Culiacan and Sinaloa, effectively, started looking for richness, and indeed got enriched during two centuries, but at around 1736 pearls started to show scarcity, so it was no longer advantageous to fish them …’ ‘some companies used five hundred or more divers. They wore rubber suits, with glass-fronted helmets, which were weighted with lead. Most of the oyster shells were taken to La Paz to be opened; often pearls of great value were found. In 1881 one was found that weighed twenty-eight carats and was sold in Paris for $10,000; two years later another was obtained that sold for $8,600.’ ‘...With the proceeds from what he took out in 1742 [Don Manuel de Ocio, a soldier from Loreto presidio], he made greater preparations for the following year, in which he obtained 127 Spanish pounds of pearls. But this catch, although abundant, was not comparable to that of 1744, which amounted to 275 pounds.’^a^	1852	Clavijero, Francisco Javier, Mexican Jesuit teacher and scholar	
‘The California Peninsula coast, from Cabo san lucas at 23° latitude until Mulegé at 27°, have the most favorable climate and water conditions, for pearl oyster preparation, and should be considered with no doubt, that today all coast of the peninsula could be a rich and abundant pleasure, if instead of procuring extermination of mother-pearl, which seems that has been the desire of divers since mid-last century, there should have been rules and authorities and army that could implement rules to subject such exploitations.’ ‘The pleasures of San Jose Island cover with some interruptions, an extension of over 80 leagues; whilst those from India are only 12 leagues and 40 to 50 those from Bahicen islands and the Persian Gulf, which are recognized for their extension and richness. It can be ensured that all coast of Baja California from Cabo San Lucas to 27° produces pearl oyster, from the East to the West of the peninsula there has been an extraction of pearls since the past, that have drawn attention for their size and sheen.’^a^	1865	José María Esteva, Mexican politician and writer	
‘… the eastern or gulf coast of the peninsula has always been the great pearl fishery of past and present history.’ ‘If all these islands, which contain immense resources in excellent harbors, in minerals, in fisheries, and in pearl-oyster banks, were joined together, they would make a district of country 100 miles long by 80 miles broad, and at a rough estimate they would make one-fifteenth of the superficies of the peninsula.’ ‘Eighty miles above San Jose is the well-known bay of La Paz, which penetrates the land to the south some twenty-five miles from Espiritu Santo Island, having a varying breadth of from six to ten miles. This is one of the safest and finest bays and harbors in the two Californias, and has been known in navigation and history for 350 years. It has been celebrated all this time for the abundance of pearl-oysters, and has produced pearls among the most valued gems of the jeweller and lapidary, and prized in the regalia of kings, emperors, and princes.’ “Tlie next harbor is the small one of the old Presidio of Loretto, which has been known since 1700. It is formed by the Coronado and Carmen Islands, and makes a fine anchorage in ordinary seasons; in its vicinity the pearl-oyster was formerly found in the greatest abundance.’ ‘the next harbor north of Loretto of value is that of Moleje, so called from an Indian camp found there by the Jesuits before 1730. It is about 20 miles deep by an average of five, and is considered the best in the gulf after La Paz. It is famous for the extent of its pearl-oyster beds, and was resorted to by the divers from the Sinaloa coast in the time of Cortez.’ ‘the large bay of Los Angeles, 180 miles above Moleje, capable as is said of holding hundreds of small vessels, has been frequently resorted to within the last ten years, and its waters and those of Angel Island abound in a peculiar species of whale and rich banks of pearl-oysters.’ ‘above Magdalena, which in the tuinter season are full to the sea. A short distance above this they found a large bay, named by them from the immense number of whales seen, Baja de Ballenas, in the position of which no two maps or charts agree. It was inhabited by myriads of sea-birds, and all kinds of shell and scale fish were found in the greatest abundance; pearl oysters were also found here, which seems to be their northern limits.’	1868	[51]	Alexander S. Taylor
‘in the lower part of the Bay of Mulege, in the Gulf of California, near Los Coyetes, pearls have been found of rare value and astonishing brilliancy. It was in this bay that Jeremiah Evans, an Englishman, towards the close of the last century, obtained those magnificent pearls, of which the collar was made for the Queen of Spain, and which evoked so much admiration at St. Cloud and Windsor Castle. In the time of the Jesuit missionaries, the pearl fishery was actively carried on, and produced great wealth to the people of Lower California.’	1883	P. L. Simmonds, Dutch writer and editor [54]	
‘La Paz, itself, is the seat of the pearl-fishing industry of California. The annual output is valued at a quarter of a million dollars, gold, and is promptly marketed in London, Paris and other great European marts. The industry is growing in size; it is in the hands of three concessionaires — the Mangara Exploration Company, Sr. G. J. Vivés and Sr. M. Carnejo, among whom the pearl-oyster sections of the coast are divided.’	1908	Arthur Walbridge North [55]	
‘The richest pearl fisheries in the Americas at the present time are those of the Gulf of California, centering around La Paz, and along the outer coast of Lower California, in México’. ‘The greater part of the shell from the La Paz fisheries is shipped to San Francisco, while most of the pearls go to Mexico city and Paris. In 1908, the production of these fisheries was valued at £1,000,000, the pearls representing £600,000, and the mother-of-pearl shell the balance. The Lower California pearls are of a great variety of shapes and colour. Some very famous pearls of history have been taken from these waters. One of the most beautiful gems of the Spanish crown is an enormous Mexican pearl, found near Loreto by a native diver, and weighing 400 grains.’	1911	*Journal of the Royal Society of Arts* [*56*]	

^a^ Quotations published in Spanish translated to English for this analysis.

### (b) Late modern period (1760–1950)

**Figure 2.**
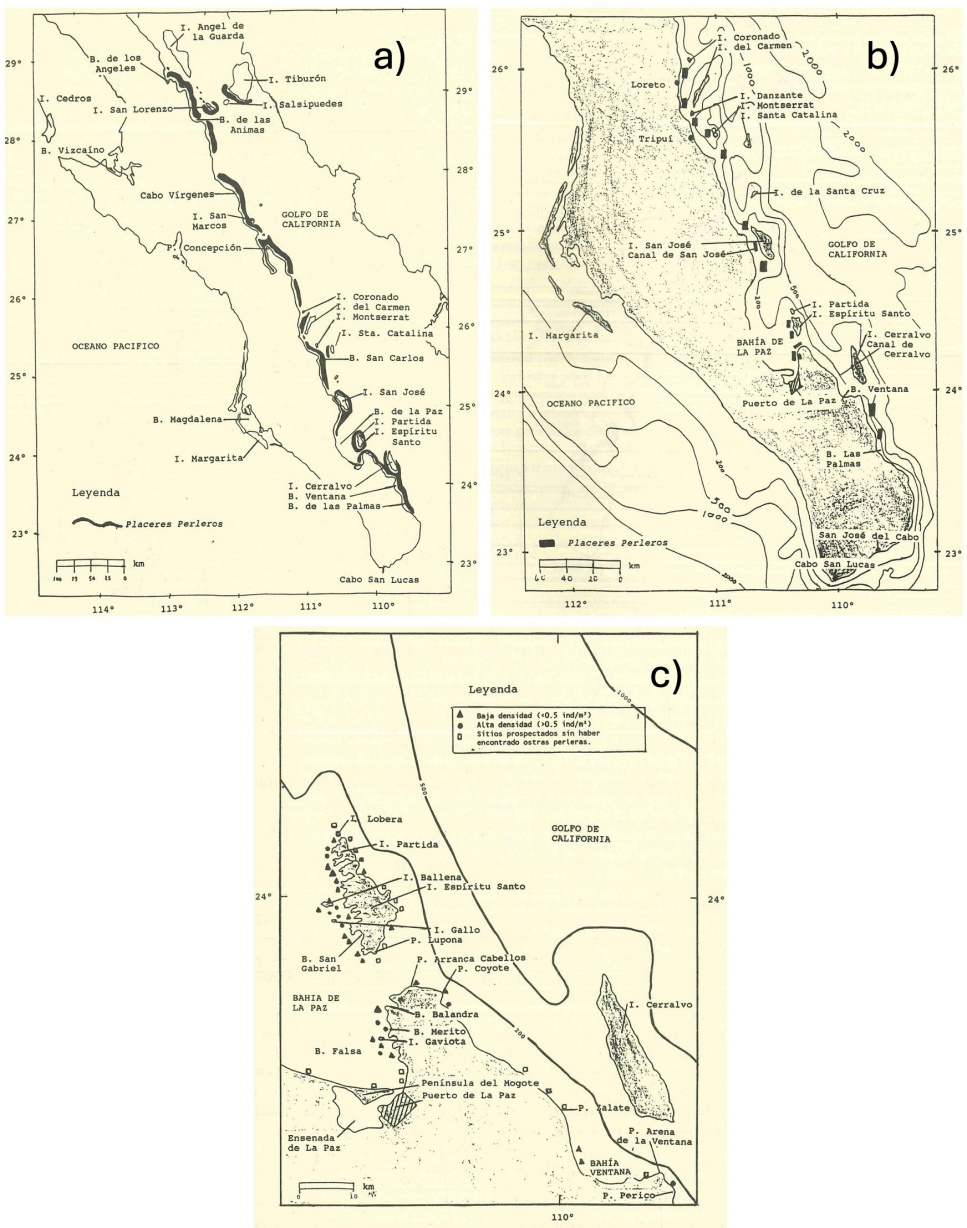
Distribution of pearl beds in the Baja peninsula from 1746 to 1992. The maps show only the distribution on the Gulf of California coast. Extracted from Cariño-Olvera [24]. This figure aggregates maps of pearl oyster bed distribution from three authors and time periods: (a) Father Stratford, 1746 (see [24]); (b) Leon Diguet, 1912 (see [24]); and (c) Monteforte & Cariño [57], guided by the previous works of Diguet (1912), Hertlein (1951) and Mosk (1931) (only La Paz Bay) (see [24]).

Maps in figure 2 show the areas of pearl oyster reefs, locally known as ‘placeres perleros’. It can be seen how these shellfish reefs extended for miles along the coast and around the islands in the Gulf of California during the mid-1700s, and by the early 1900s, these were decimated to isolated ‘patches’. In the late 1900s, Monteforte & Cariño [57] undertook field studies to estimate the presence and population density of the two species (*P. mazatlanica* and *Pteria sterna*) of pearl oysters around Espiritu Santo Island and La Paz Bay, where their site selection was informed by the works of previous studies. The authors found dispersed areas of high and low abundance, and an absence of *P. sterna* in some of the sites explored, evidencing impoverishment in these populations.

**Figure 3.**
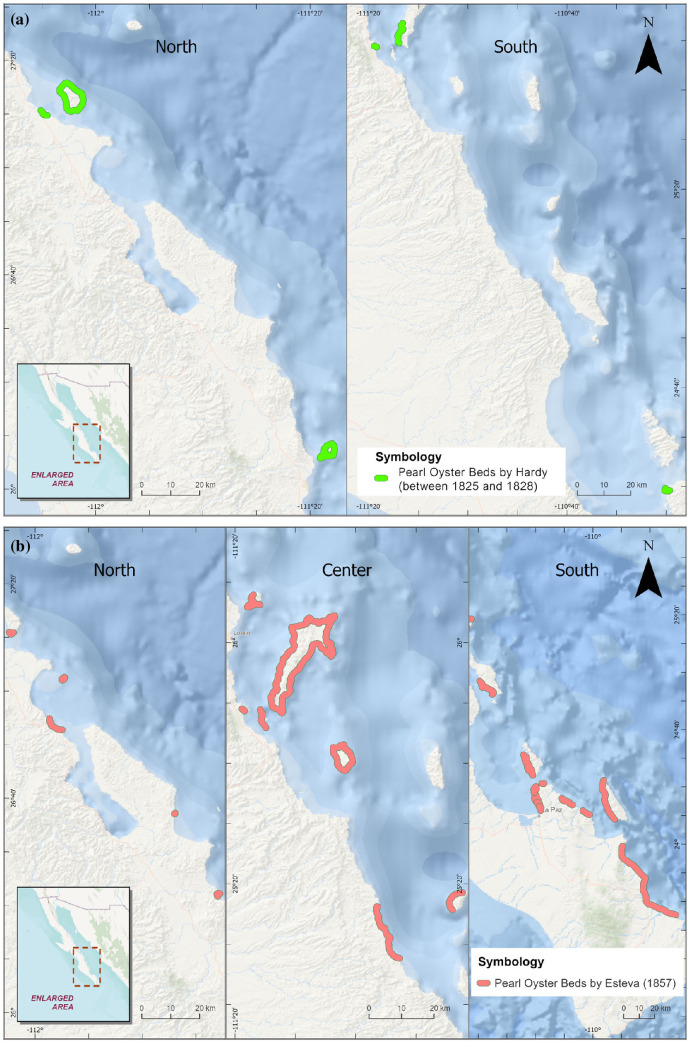
Digitized maps from descriptions of the distribution of oyster beds. (a) Location of pearl oyster beds in the north, centre and south of Baja California Sur, digitized from government report descriptions of the pearl fishery in 1857, written by José María Esteva, politician and writer. (b) Pearl oyster beds around the Loreto area, digitized from a book published in 1977, by English Lieutenant Hardy, Robert William who explored the Gulf of California between 1826 and 1827.

Several authors provide detailed and general descriptions of the abundance and distribution of pearl oyster beds during different periods of time. It is interesting that some of the ‘placeres’ were widely recognized, such as La Paz, Pichilingue and Loreto areas, while others reflect knowledge that was limited to some explorers or more ambitious merchants or businessmen who ventured on longer trips with higher risks, including the oyster pearl beds around Tiburón Island under the management of Seri indigenous group. Leese [58] reported that in 1855 pearl diving yielded 6 90 000 pounds of shell (valued at 6 cents), accounting for $41 000 and the pearls obtained sold for $23 800. José María Esteva, who held an interim charge in the Baja peninsula government did thorough research on the pearl fishery to map the pearl oyster reefs in three regions in Baja California Sur (figure 3), as well as detailed documentation of the business of the pearl fishery in La Paz, Mulegé and Loreto Districts, including the cost and revenues of this activity which in 1856 accounted for a yield worth over $33 437, employing at least 305 divers. Accompanying the government report, Esteva adds an official decree with management measures for the fishery, including the assignment of harvest areas and a rotation system, indicating his concern about this resource condition [26]. Monteforte & Cariño [57] estimated that in 1904 the extraction of pearl oysters in the Gulf of California reached 2500 million shells.

### (c) Contemporary history (1950 to present)

**Figure 4.**
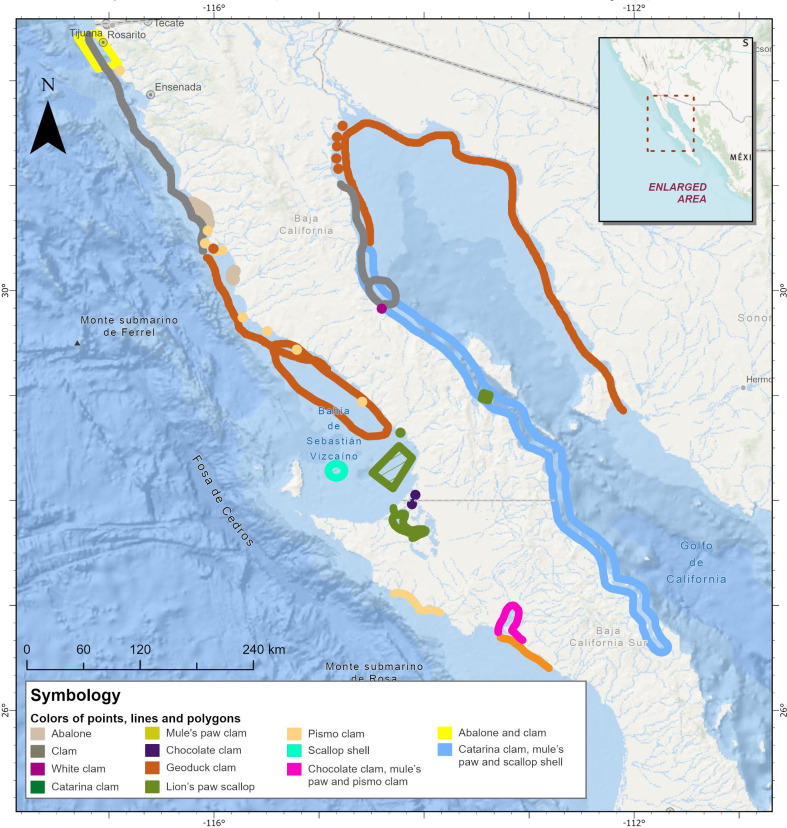
Results of participatory mapping of historic bivalve bank distribution. Geospatial data from participatory mapping exercises for the Baja peninsula. Participants were asked to mark areas where they considered that bivalve banks had been present historically, regardless of their current status. Note that this exercise was done only with stakeholders from Baja California, so we have a gap of information for Baja California Sur. Participants included abalone due to its historic commercial and ecological importance. Participants drew points, lines and polygons which denote bivalve presence.

Participants drew areas for historic bivalve banks, highlighting 11 groups of commercially important species: pismo clam (*Tivela stultorum*), Pacific lion’s paw (*Nodipecten subnodosus*), geoduck clam (*Panopea generosa*, *P. globosa*), chocolata clam (*M. squalida*, *M. aurantiaca*), chione clam (*C. undatella*, *C. californiensis*, *Chionista fluctifraga*, *Chionopsis gnidia*), mule’s paw clam (*Larkinia grandis*, *L. multicostata*, *Anadara tuberculosa*)*,* concave scallop (*Euvola vogdesi*), catarina clam, white clam (*Dosinia ponderosa*), penshell scallop (*Pinna rugosa*, *Atrina maura*, *A. tuberculosa*, *A. oldroydii*) and mussels (*Mytilus californianus*, *M. galloprovinsialis*, *Modiolus capax*, *Mytella guyanensis*, *M. edulis*, *M. strigata*). Both sides of the Baja peninsula were recorded as being completely covered, reflected in points, lines or polygons (figure 4).

Building from the mapping exercises and multi-stakeholder workshops, The Nature Conservancy, in collaboration with IMIPAS, developed a smart siting tool for bivalve and finfish groups that can inform future site selection for aquaculture farms, based on environmental, infrastructure, natural resource and the participatory mapping information which supported the generation of a suitability analysis, now hosted online at #/.

A total of 199 aquaculture leases (66 from Baja California and 133 from Baja California Sur) offshore and nearshore, are currently permitted. The Pacific coast shows a significantly higher density of leases than the Gulf of California. A single lease can authorize the culture of multiple species and culturing gear, with the Pacific oyster being the species most cultured, amongst a list of at least other 17 bivalve species including clams, penshells, mussels and oysters.

A total of 558 fishing permits (553 commercial, two research and three fishing concessions) for at least 30 species of bivalves currently operate in the region. While some permits include the geographic coordinates of fishing areas, others mention a region, for example, ‘Federal jurisdiction waters of the Gulf of California coast, adjacent to the state of Baja California Sur, in front of the Municipality of La Paz’.

## 4. Discussion

**Figure 5.**
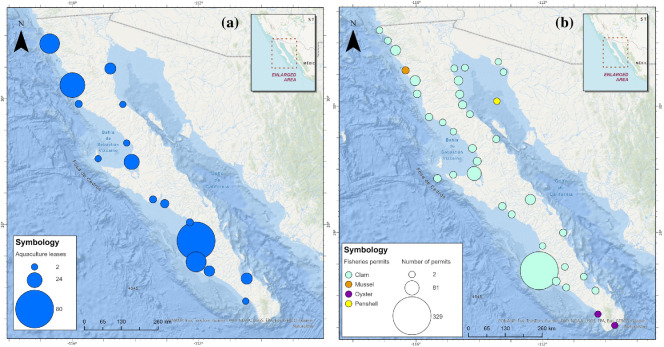
Current distribution of (a) aquaculture leases (permits and concessions), and (b) fishery permits for bivalve species.

General bivalve populations are poorly understood and have been subject to mismanagement and overexploitation, and there is disregard for their ecological function as reef builders [1]. However, there is an opportunity to invest in large-scale restoration efforts and restorative aquaculture, as these opportunities present a promising pathway that can contribute to rebuilding services from these ecosystem engineers. Our study shows that biogenic reefs have been critical for species in the Baja peninsula, ecologically and socially, since prehistoric times and through the development of modern society. Data indicate their presence and diversity since the Mesozoic Era, and more recently the Miocene and Pliocene epochs, over 5 million years ago, where fossil records show over 34 species of bivalves in some localized areas like La Paz, and 14 pectinid and oyster species in the Loreto Basin. This includes *Crassostrea californica osunai* which is likely to have been endemic to Baja California Sur [4,59]. Fishing permits for bivalves in the Baja peninsula allow the harvest of at least 30 species including clams, penshell, oyster (*C. costeziensis*, *C. virginica*) and mussels*,* with clams and oysters included among the main fisheries for Baja California and Baja California Sur which rank third and fourth place, respectively, in both fisheries volume and value nationally ([38]; figure 5b). Despite their wide distribution and importance, there is vast documentation of the overexploitation that these systems have suffered, and reefs created by several of these species no longer exist at the scale they once did. Studies in northern Baja show declines in native species and changes in intertidal bivalve communities over the last 30−50 years [60] and the National Fisheries Chart indicates significant declines of historic fisheries production in catarina clam, chocolate clam, Pacific thorny oyster (*Spondylus crassisquama*), geoduck clam, mule’s paw clam, chione clam, penshell and the California mussel; for most of these species, the status is overexploited or harvested at their maximum sustainable yield [61] and some populations have collapsed, such as the concave scallop. In some species, landings declines have led to temporal or permanent fishery closures, and some have been listed as protected species, such as the pismo clam and mother-of-pearl oyster ([62,63]).

Besides unsustainable harvest, the condition of these resources is exacerbated by additional stressors that can result in a cumulative impact, including disease, pollution, climate change or climatic variations [64], invasive species like ascidians and boring polychaetes [65,66], among others. Additionally, some species such as catarina clam naturally present highly fluctuating populations, although the factors behind this volatility are unknown [67]. Mexico and the Gulf of California have ranked highly in global analyses of the potential to expand low trophic level aquaculture owing to the country’s low production volumes by the area of Exclusive Economic Zone, a number of species currently under cultivation only being produced to a low level, and the opportunity to capitalize on restorative aquaculture practices to generate positive commercial and environmental outcomes [11,68].

Oysters and clam production contribute between 20 and 38% of the Baja California and Baja California Sur total aquaculture production, respectively. Looking at the distribution of aquaculture leases and considering the over 3000 km coastline of the Baja peninsula, it can be recognized that the development of aquaculture in this region has been limited, compared with other areas with high hydric potential. Although over 17 species of bivalves are included in the aquaculture leases, there is a strong focus on the Pacific oyster partly because of the installed infrastructure and capacity, and market demand, resulting in a missed opportunity for investing and developing other species. Despite a profound knowledge of biology and hatchery production for native species, only few commercial aquaculture operations have been established and laboratory production is still challenging [64]. Extensive programmes and research studies have operated for penshell, concave scallop, catarina clam and lion’s paw clam, among other species; nevertheless, challenges related to spat supply (both for laboratory and collection in the wild) and the market remain [64]. However, efforts of successful wild seed collection, grow out and harvest for catarina clam, penshell, Cortez oyster and other native species in La Paz Bay and Bahía Magdalena provide promising examples for future investment and development around native species restoration ([36,69–71); A. Gorosave 2024, personal communication).

Looking into the future, existing climate change scenarios or projections for a wide range of species, including penshell, chocolata clam, Cortez oyster and pearl oyster [72], provide insights to potential distribution of these bivalve populations in the Gulf of California, suggesting that the region will continue to provide a highly suitable area for the development and successful culture of these important species that could contribute to restoration of shellfish ecosystems in the region [73].

Key opportunities for expanding the aquaculture industry include financing mechanisms and support for small-scale farmers, research and development of native species, capacity building and infrastructure, hatchery spat production, market strategies and development/update of aquaculture legislation [39,64]. Public policy can support a nature-positive transition in the national aquatic food production system, recognizing multisectoral collaboration, prioritizing investment in research, infrastructure and development, and promoting equitable and inclusive opportunities to local communities [32,74]. Moreover, defining the types of aquaculture can help us understand their objectives and scope, supporting industry and government to advance sustainable aquaculture. For example, restorative aquaculture occurs when commercial or subsistence aquaculture provides direct ecological benefits to the environment, with the potential to generate net positive environmental outcomes [75]. Recognizing such concepts and their benefits can lead to the development of policy approaches that encourage these types of practices through compensation schemes for farmers, such as subsidies and fiscal incentives, including the reduction/exemption of taxes, subsidies for the application of CO_2_ reducing technologies, and even accelerating the permitting processes.

Consistent with the literature on the abundance and distribution of bivalve banks in the past, perception among farmers and key stakeholders today reaffirm that historically, bivalve banks were large, numerous and diverse along the coast of the Baja peninsula and even the mainland. We elicited this understanding by collating historical data and anecdotal information, and through a participatory process focused on spatial mapping. Participatory processes are critical to incorporate community knowledge and values into decision-making, specifically local spatial knowledge [42,76]. They can also improve the accuracy, applicability and accessibility of spatial data, increasing engagement and long-term support from user groups for the process and tools produced [42,76,77]. We observed this heightened engagement with the overall process of spatial planning for aquaculture, receiving positive comments on both the method and the value of the broader work after each meeting and in follow-up discussions. Participatory mapping in marine environments has been used to support the development of conservation areas (e.g. [78] and successful examples for fisheries management in the Gulf of California include understanding the spatio-temporal dynamics of the warrior swimming crab fleets [79] and design of Fishing Refuge Zones [80]; however, participation has been lower in the formation of collaborative approaches to industry development, such as siting of new aquaculture activities. Spatial planning and site selection for aquaculture often relies on remotely sensed spatial data (e.g. satellite-derived characteristics and datasets). We found that the participatory process was also a way to gather insights on key information and data needs to understand and calibrate the suitability of areas for growing under current conditions, and to begin considering the accuracy and interoperability of remotely sensed data. For example, some areas that ranked as ‘highly suitable’ in our analysis were considered by participants to have a low suitability due to wave height and strong currents, questioning why there might be a mismatch in this interpretation and whether any gaps or issues with the spatial data might exist. It is interesting to note the data overlap from all time periods, potentially indicating highly suitable areas for bivalve aquaculture throughout the Baja peninsula, consistent with priority fisheries and aquaculture areas in state development plans [67,81].

An aspect that warrants consideration is the site selection and compatibility between competing sectors. Despite perceived conflicting interests, aquaculture can go hand by hand with other industries and conservation efforts. The San Ignacio Lagoon, which is a Marine Protected Area, includes a highly successful Pacific oyster farm, providing sheltered estuarine areas and surveillance that supports its operation. Other examples are La Paz and Magdalena Bay, where groups of organized fishers have started touristic routes to show their aquaculture farms, provide an oyster tasting and educate tourists around the farming practices [70,71]. The fishing sector is of special interest to aquaculture as they may both depend on the success of the other, especially for species that rely on the collection of wild spat and, therefore, the success of their culture is linked to the management of the species fishery. On the other hand, aquaculture could contribute to habitat provision for fish and invertebrates, and the repopulation of bivalve banks that are adjacent to aquaculture operations.

Spatial planning and siting of aquaculture can contribute to a smart growth of this sector and to reduce conflict between competing industries, but it can also help maximize benefits and minimize economic and social costs. Collaboration with other sectors could potentially resolve conflicts in the sharing of land, water and other natural resources, but it requires important organization efforts [74].

Our study describes the widespread systematic loss of shellfish ecosystems and species abundance along the Baja peninsula and identifies an opportunity for conservation efforts and restoration to be coupled with an emerging interest in aquaculture. However, in furthering these approaches attention must be given to the current context. While some species may have occurred historically in areas, ecological conditions may no longer be favourable for their survival or growth, and restoration or aquaculture efforts in these areas may therefore have limited success [82]. These efforts should proceed with further research on species-specific tolerances that can monitor restored stock for mortality or other unforeseen events, such as high rates of disease. Historical and local ecological knowledge at a local scale will also help to guide the potential efficacy of these efforts. While our work adds to understanding of the past distribution of shellfish along the Baja peninsula and informs potential site selection for restoration work, the scale of our analysis is regional and should be supplemented with additional local ecological knowledge from individuals and communities. Moreover, the literature sources we used represent a small subsection of the available material and more detailed and targeted research could improve the overall data mapped and its resolution. Potential tradeoffs as well as synergies between aquaculture and other sectors and industries, such as conservation and fisheries, should likewise be considered, prior to restoration and aquaculture efforts and managed thereafter. These industries often experience similar challenges and identifying synergistic effects and opportunities in resource rights, risk reduction and the development and maintenance of supply chains or new markets, especially for native species can bring benefits to all user groups, reducing potential conflicts and making benefits equitable within and across communities [83].

## 5. Conclusion

Information about historic presence and distribution of bivalves can provide useful insights to better channel and prioritize investment for future restoration efforts, and the sustainable use of resources through existing industries such as fisheries and aquaculture [84] as well as emerging models of ecosystem-centred production [85]. Although some systems and species found along the Baja peninsula have undergone transformational changes and will never return to their pristine state, some systems may be resilient enough to not only withstand such dramatic changes, but to also recover to some extent once anthropogenic stressors have been reduced or removed. Changed ecological baselines should not necessarily be perceived as doomed, but rather, they provide critical indicators that help guide restoration efforts of species that once flourished under certain environments. Where past ecological baselines have been identified and used as an informant of the need and design for restoration work, they have been transformative in motivating large-scale environmental repair, and identifying novel solutions that bring together communities, government and industry on successful restoration work [12]. Our work contributes important historical baselines for critical marine ecosystems in the Gulf of California and provides the data and justification needed to move forward with conservation efforts and restorative aquaculture.

## Data Availability

Supplementary material is available online [86].
